# The Non-Heredity of Inebriety

**Published:** 1896-01

**Authors:** 


### BOOK NOTICES.

The Non-Heredity of Inebriety, by Leslie E. Keeley, M. D., LL. D., is the title of a timely volume now in the press of S. C. Griggs & Co., of Chicago.

The author endeavors to show that inebriety is a disease, and that it, as well as other diseases, is not hereditary. The work is said to differ from others on inebriety in its application of the doctrines of the variation of species and natural selection to cell life, thus showing the causes and nature of disease, its modern scientific treatment, and the philosophy of immunity to disease in general, and inebriety in particular—all in language within the comprehension of the general reader. The international reputation of the author as an original investigator in matters pertaining to inebriety should make this work of more than ordinary value to scientists, the medical profession, and to all who are, by legislation or otherwise, endeavoring to correct the evils of intemperance.

